# Effectiveness of Booster and Influenza Vaccines against COVID-19 among Healthcare Workers, Taiwan

**DOI:** 10.3201/eid2810.221134

**Published:** 2022-10

**Authors:** Jun Yi Sim, Ping-Sheng Wu, Ching-Feng Cheng, You-Chen Chao, Chun-Hsien Yu

**Affiliations:** Taipei Tzu Chi Hospital, Buddhist Tzu Chi Medical Foundation, Taipei, Taiwan; and Tzu Chi University, Hualien, Taiwan

**Keywords:** COVID-19, respiratory infections, influenza, severe acute respiratory syndrome coronavirus 2, SARS-CoV-2, SARS, coronavirus disease, zoonoses, viruses, coronavirus, ChAdOx1 nCoV-19, 2019-nCoV vaccine mRNA-1273, BNT162 vaccine, MVC-COV1901 vaccine, healthcare personnel, Taiwan

## Abstract

Among previously uninfected healthcare workers in Taiwan, mRNA COVID-19 booster vaccine was associated with lower odds of COVID-19 after primary recombinant vaccine. Symptom-triggered testing revealed that tetravalent influenza vaccine was associated with higher odds of SARS-CoV-2 infection. COVID-19 vaccination continues to be most effective against SARS-CoV-2.

Border control, contact tracing, and adherence to nonpharmaceutical interventions enabled Taiwan to contain COVID-19 for >2 years (*1*). From the beginning of the pandemic in 2020 through March 31, 2022, Taiwan had just 16,224 domestic COVID-19 cases, an incidence of 0.07% for a population of 23.6 million (*2*). In this backdrop, we found no COVID-19 cases among healthcare workers (HCWs) at Taipei Tzu Chi Hospital, Taipei, Taiwan, through April 10, 2022, despite symptom monitoring and surveillance. 

Meanwhile, to overcome vaccine shortages and hesitancy among adults in Taiwan, homologous and heterologous regimens of the adenoviral vector vaccine ChAdOx1-S/nCoV-19 (AstraZeneca, https://www.astrazeneca.com), the adjuvanted subunit protein vaccine MVC-COV1901 (Medigen, https://www.medigenvac.com), and the mRNA vaccines mRNA-1273 (Moderna, https://www.modernatx.com) and BNT162b2 (Pfizer-BioNTech, https://www.pfizer.com) were used widely. All vaccines were given in a 2-dose primary series and for a 1-dose booster, except for ChAdOx-S/nCoV-19 (*3*).

To evaluate effectiveness of COVID-19 booster vaccines and the 2021–22 tetravalent seasonal influenza vaccine against COVID-19 during an Omicron variant–predominant surge, we conducted a retrospective study of HCW vaccination at Taipei Tzu Chi Hospital. We obtained an employee list with vaccination data and COVID-19 surveillance reports from the hospital for April 10–June 10, 2022. During this period, the hospital tested HCWs in 2 groups: the routine testing group comprised emergency department and COVID-19 ward staff who received regular, weekly testing; the symptom-triggered group comprised staff who were tested whenever symptoms developed or after a high-risk exposure. Nasopharyngeal swab samples were collected by professionals and tested for SARS-CoV-2 by reverse transcription PCR (RT-PCR) using a previously described RT-PCR protocol (*4*) or by Panbio rapid antigen test (Abbott, https://www.abbott.com). This study received approval from the Taipei Tzu Chi Hospital institutional review board with waiver for informed consent because the study used previously collected data (approval no. 11-X-106). 

We compared data by using 2-tailed χ^2^ and Kruskal-Wallis tests and considered p<0.05 statistically significant. We used a multivariate logistic regression model to assess the relationship between SARS-CoV-2 infection during April 10–June 10, 2022, and age, sex, work sector, COVID-19 booster vaccine, and seasonal influenza vaccination. We performed all analyses in SPSS Statistics 25.0 (IBM, https://www.ibm.com).

The employment list included a total of 2,544 HCWs; we excluded 348 (13.7%) staff who were outsourced, who were on extended leave, or who had resigned. Of the remaining 2,196 HCWs, 453 (20.6%) tested SARS-CoV-2 positive during the study period (Figure). COVID-19 incidence was highest (35.3%) among housekeeping staff and lowest (13.5%) among medical staff (Table). All COVID-19–positive HCWs experienced mild symptoms; none required intensive care. 

**Figure:**
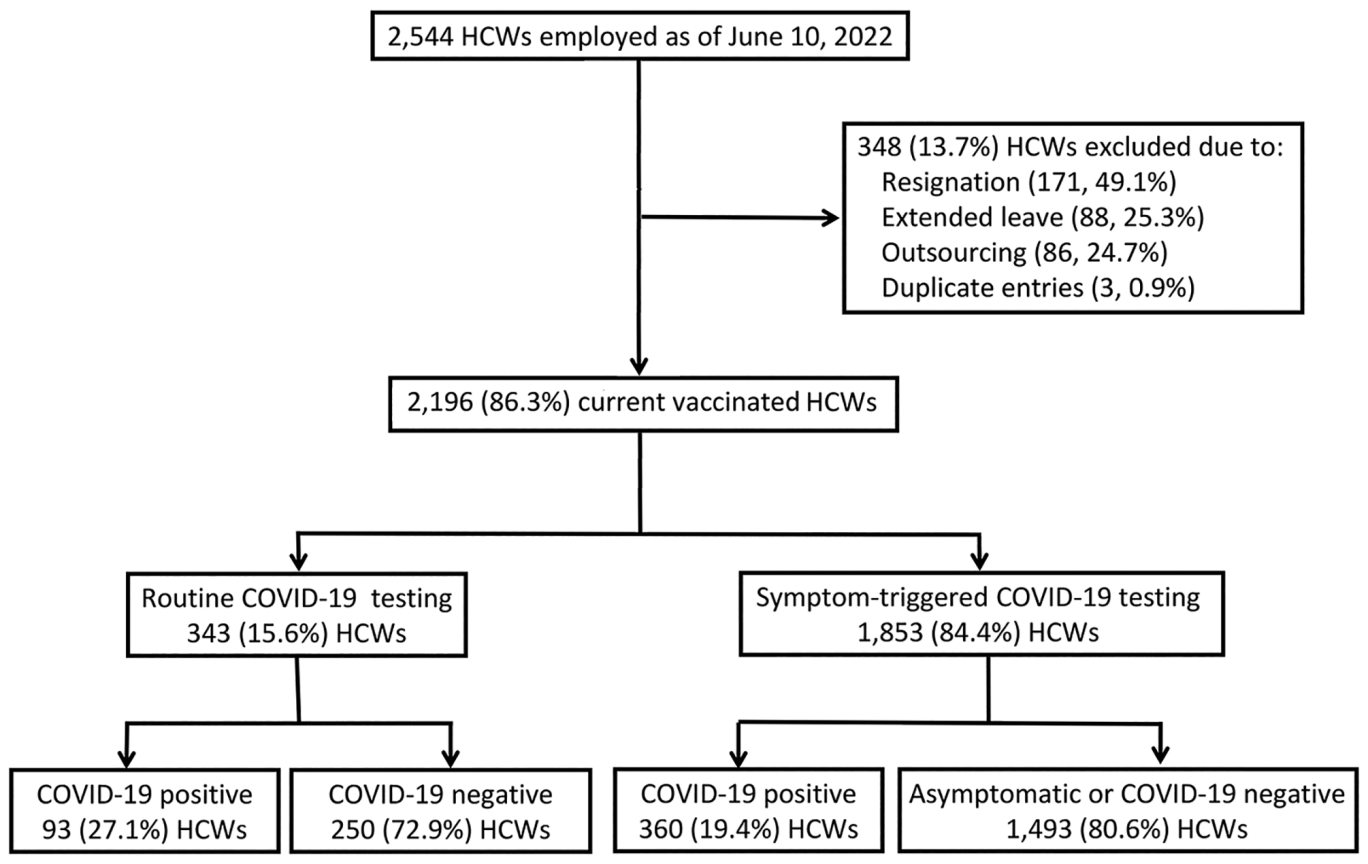
Worker exclusion and testing in a study of the effectiveness of booster and influenza vaccines against COVID-19 among healthcare workers, Taipei Tzu Chi Hospital, Taipei, Taiwan. Employment, vaccination, and testing data for April 10–June 10, 2022, were provided by the hospital’s Human Resource Office and corroborated by the Occupational Safety and Health Administration Office and the hospital’s Center for Infection Control. Workers in the routine testing group were tested weekly by reverse transcription PCR or rapid antigen test; workers in the symptom-triggered testing group were tested if COVID-19 symptoms developed or after they were exposed to COVID-19 cases. HCW, healthcare worker.

**Table:** Characteristics of 2,196 healthcare workers tested for SARS-CoV-2 after receiving COVID-19 booster and influenza vaccines, Taipei Tzu Chi Hospital, Taipei, Taiwan*

Characteristics	Regular testing, n = 343†					Symptom-triggered testing, n = 1,853			
	Positive, no. (%)	Negative, no. (%)	p value	OR (95% CI)		Positive, no. (%)	Negative or no symptom, no. (%)	p value	OR (95% CI)
Total	93 (100)	250 (100)	NA	NA		360 (100)	1,493 (100)	NA	NA
Sex			0.051					0.084	
F	67 (72.0)	205 (82.0)	NA	1.4 (0.7–3.1)		278 (77.2)	1,085 (72.7)	NA	1.0 (0.7–1.4)
M	26 (28.0)	45 (18.0)	NA	Referent		82 (22.8)	408 (27.3)	NA	Referent
Age range, y			0.377					0.345	
71–80	1 (1.1)	0	NA	NA		1 (0.3)	8 (0.5)	NA	0.7 (0.1–6.0)
61–70	1 (1.1)	4 (1.6)	NA	0.7 (0.1–6.6)		6 (1.7)	55 (3.7)	NA	0.5 (0.2–1.3)
51–60	3 (3.2)	18 (7.2)	NA	0.5 (0.1–2.0)		33 (9.2)	160 (10.7)	NA	0.9 (0.6–1.3)
41–50	15 (16.1)	39 (15.6)	NA	0.9 (0.4–1.9)		93 (25.8)	362 (24.2)	NA	1.0 (0.7–1.4)
31–40	29 (31.2)	63 (25.2)	NA	1.1 (0.6–2.1)		92 (25.6)	391 (26.2)	NA	1.0 (0.7–1.3)
21–30	44 (47.3)	126 (50.4)	NA	Referent		135 (37.5)	517 (34.6)	NA	Referent
Work sector			<0.001					<0.001	
Nursing	52 (55.9)	175 (70.0)	NA	0.1 (0.1–0.4)		170 (47.2)	641 (42.9)	NA	0.9 (0.6–1.2)
Medical	15 (11.3)	37 (14.8)	NA	0.2 (0.1–0.5)		39 (10.8)	309 (20.7)	NA	0.5 (0.3–0.8)
Technical	6 (6.5)	9 (3.6)	NA	0.3 (0.1–1.1)		43 (11.9)	197 (13.2)	NA	0.8 (0.5–1.2)
Laboratory, pharmacy	3 (3.2)	20 (8.0)	NA	0.1 (0.0–0.3)		32 (8.9)	100 (6.7)	NA	1.1 (0.7–1.8)
Housekeeping	0	2 (0.8)	NA	NA		6 (1.7)	9 (0.6)	NA	2.8 (0.9–8.6)
Administration	17 (18.3)	7 (2.8)	NA	Referent		70 (19.4)	237 (15.9)	NA	Referent
No. COVID-19 vaccine doses			0.065					<0.001	
3	88 (94.6)	246 (98.4)	NA	0.2 (0.0–0.8)		319 (88.6)	1,416 (94.8)	NA	0.4 (0.2–0.6)
2	5 (5.4)	4 (1.6)	NA	Referent		38 (10.6)	73 (4.9)	NA	Referent
1	0	0	NA	NA		0	0	NA	NA
0	0	0	NA	NA		3 (0.8)	4 (0.3)	NA	NA
COVID-19 primary series‡			0.442					0.348	
Viral vector + viral vector	70 (75.3)	193 (77.2)	NA	NA		270 (75.0)	1,091 (73.1)	NA	NA
Viral vector + mRNA	7 (7.5)	27 (10.8)	NA	NA		45 (12.5)	189 (12.7)	NA	NA
mRNA + mRNA	15 (16.1)	30 (12.0)	NA	NA		41 (11.4)	206 (13.8)	NA	NA
Protein subunit + protein subunit	0	0	NA	NA		0	2 (0.1)	NA	NA
COVID-19 booster§			1.00					0.145	
mRNA	86 (92.5)	243 (97.2)	NA	NA		317 (88.1)	1,387 (92.9)	NA	NA
Protein subunit	0	2 (0.8)	NA	NA		2 (0.5)	27 (1.8)	NA	NA
Booster vaccine date¶			0.111					<0.001	
December 2021	23 (24.7)	68 (27.2)	NA	NA		70 (19.4)	319 (21.4)	NA	NA
January 2022	55 (59.1)	164 (65.6)	NA	NA		219 (60.8)	999 (66.9)	NA	NA
February 2022	4 (4.3)	4 (1.6)	NA	NA		17 (4.7)	35 (2.3)	NA	NA
March 2022	3 (3.2)	7 (2.8)	NA	NA		5 (1.4)	32 (2.1)	NA	NA
April 2022	0	3 (1.2)	NA	NA		6 (1.7)	24 (1.6)	NA	NA
May 2022	1 (1.1)	0	NA	NA		0	4 (0.3)	NA	NA
Tetravalent influenza vaccine, 2021–22 season			0.297					0.016	
Vaccinated	68 (73.1)	167 (66.8)	NA	1.5 (0.8–2.7)		265 (73.6)	1,001 (67.0)	NA	1.5 (1.1–2.0)
Not vaccinated	25 (26.9)	83 (33.2)	NA	Referent		95 (26.4)	492 (33.0)	NA	Referent

*p values calculated by using χ^2^ test; OR and 95% CI calculated by using multinomial logistic regression. NA, not applicable; OR, odds ratio. †For age 71–80 y and housekeepers of the regularly tested subgroup, estimates were not shown because the groups were too small. ‡Excluding 7 unvaccinated workers and 3 workers who did not report vaccine type. Data available for a total of 2,186 healthcare workers; regular testing subgroup included 92 positive cases and 250 negative cases; symptom-triggered testing subgroup included 356 positive cases and 1,488 negative cases; thus, percentages do not add up to 100%. §Excluding 7 unvaccinated workers, 120 workers who did not receive a booster vaccine, and 5 workers who did not report booster vaccine type. Data available for a total of 2,064 healthcare workers; regular testing subgroup included 86 positive cases and 245 negative cases; symptom-triggered testing subgroup included 319 positive cases and 1,414 negative cases; thus, percentages do not add up to 100%. ¶Excluding 7 unvaccinated workers, 120 workers who did not receive a booster vaccine, and 7 workers who did not report month of booster vaccine. Data available for a total of 2,062 healthcare workers; regular testing subgroup included 86 positive cases and 246 negative cases; symptom-triggered testing subgroup included 317 positive cases and 1,413 negative cases; thus, percentages do not add up to 100%.

COVID-19 vaccine uptake was 99.7% for primary series and 94.5% for booster doses; booster uptake was highest (94.9%) among technicians and lowest (92.7%) among administrators. Influenza vaccine uptake was 68.4%, highest (74.3%) among nurses and lowest (52.9%) among housekeeping staff. 

Compared with HCWs who had symptom-triggered testing, regularly tested HCWs were younger (median age 31.0 years, interquartile range [IQR] 26.0–40.0 years, vs. 36.0 years, IQR 28.0–45.5 years; p<0.001). Regularly tested HCWs also were more likely to be female (79.3% vs. 73.6%; p = 0.026), have had received booster vaccination (97.4% vs. 93.7%; p = 0.005), and have tested COVID-19–positive (27.1% vs. 19.4%; p = 0.002). Influenza vaccine uptake and types of primary and booster regimens were not greatly different for either subgroup (Table).

Regression analyses identified receiving booster vaccination and being medical staff were also associated with lower odds of COVID-19 for both testing subgroups. Tetravalent influenza vaccination was associated with higher odds of COVID-19, although we observed statistically significant results only for HCWs who underwent symptom-triggered testing (Table).

Effectiveness of primary ChAdOx-S/nCoV-19 series coupled with mRNA booster is limited because some countries suspended use of ChAdOx-S/nCoV-19 because of thromboembolic concerns (*5*,*6*). However, our study provides real-world insights into effectiveness of mRNA booster after primary homologous and heterologous ChAdOx-s/nCoV-19 regimens. Our results showed a booster dose was associated with much lower odds of COVID-19 among HCWs in both the routine and symptom-triggered testing subgroups compared with HCWs having no booster. These findings are similar to observations of fewer COVID-19 infections among BNT162b2-boosted HCWs (*7*) and observed effectiveness of mRNA-1273 (47.3%) and BNT162b2 (49.4%) boosters against symptomatic Omicron infection (*8*).

A meta-analysis suggested reduced COVID-19 susceptibility with influenza vaccination for the general population but not HCWs (*9*). However, we observed a statistically significant increase in odds for COVID-19 among HCWs in the symptom-triggered testing group but not the routine testing group (p<0.001). The effect of influenza vaccines against COVID-19 among HCWs remains to be elucidated.

Study limitations include lack of universal testing and use of self-reported symptoms, which might have missed some cases. Also, vaccinated HCWs can be asymptomatically infected (*10*); hence, COVID-19 infections might be underreported in our study. Causality could not be inferred due to the study’s observational nature. We also did not account for individual behaviors and household exposures. Nevertheless, our study highlights the benefits of booster COVID-19 vaccination and its effectiveness against SARS-CoV-2 among HCWs.
